# Editorial: Reproducibility and rigour in infectious diseases - surveillance, prevention and treatment

**DOI:** 10.3389/fmed.2023.1294969

**Published:** 2023-10-11

**Authors:** Francisco Westermeier, Nuno Sepúlveda

**Affiliations:** ^1^Institute of Biomedical Science, Department of Health Studies, FH Joanneum University of Applied Sciences, Graz, Austria; ^2^Centro Integrativo de Biología y Química Aplicada (CIBQA), Universidad Bernardo O'Higgins, Santiago, Chile; ^3^Faculty of Mathematics and Information Science, Warsaw University of Technology, Warsaw, Poland; ^4^CEAUL – Centro de Estatística e Aplicações da Universidade de Lisboa, Lisbon, Portugal

**Keywords:** scientific method, deficient statistical methods, COVID-19, long-COVID, degree of scientific rigor

## Background

The US National Institutes of Health defines scientific rigor as “the strict application of the scientific method to ensure robust and unbiased experimental design, methodology, analysis, interpretation and reporting of results” (1). Scientific rigor can be conveniently divided into six levels (2): (i) insidious (or unethical) rigor, where the researcher fakes data and research findings; (ii) creative rigor, where the researcher reports data that are only consistent with his/her working hypothesis; (iii) careless rigor, where the researcher applies rigor only when asked or where it is easy to apply; (iv) selective rigor, where rigor is applied only to scientific procedures that prior experience dictates to be strictly necessary; (v) careful rigor, where the researcher seeks to avoid misleading and biased results by adhering to standards outlined by funding agencies, journals, or publishers; (vi) enduring rigor, where findings are independently replicated at different levels. Clearly, the application of insidious and creative rigors is an undesirable source of irreproducible findings. In contrast, careful and enduring rigor have a high chance of leading to reproducible findings. In theory, the research community should aim at the research standards and rules dictated by these high levels of rigor.

In Mathematics and related disciplines, research is often thought to operate at the level of careful and enduring rigor due to the logical, coherent, and deductive nature of the mathematical practice. The underlying assumption is that “*the use of formal language, axioms and strict rules of inference in mathematics leads to unquestionable mathematical knowledge*” (3); note that the infallibility view of mathematical research has been challenged by empirical data (4). In contrast, medical and public health research on infectious diseases is often conducted at a fast pace because of its potential impact on addressing dramatic humanitarian crises, such as the Cholera outbreak in Yemen (5), the Ebola Virus epidemic in West Africa (6), the spread of the Zika Virus in the Americas (7) and, of course, the devastating COVID-19 pandemic (8, 9). The emergency nature of this research exerts additional pressure on a research community that is already living under a “publish-or-perish” mindset due to the limited number of university positions (10). To make the situation worse, the acceptance rate of reviewers willing to review submitted manuscripts is declining over time (11), a trend that has been interpreted as “reviewers' fatigue.” These conditions promote an unintended reduction in the scientific rigor.

In this scenario, we called for participation in a Research Topic dedicated to collect opinions and perspectives on scientific rigor and reproducibility in medical and public health research studies on infectious diseases. The call for participation was held open from 18/11/2021 to 30/11/2022, a time window that was still under the strong influence of the COVID-19 pandemic. Unsurprisingly, almost all the published papers referred to comments on this disease. Below we provided a brief overview of the 6 published papers.

## Overview of published research studies and opinions

Retrospective analysis of the COVID-19 literature revealed three waves of Research Topics during the pandemic (12). Initially, research efforts focused on the basic epidemiologic and clinical characterization of the disease. This effort then shifted to questions about COVID-19 herd immunity, serologic testing, and asymptomatic characterization. In the latter stages of the pandemic, research shifted to vaccines and their therapeutic efficacy and comparability. It also aimed at predicting new infection waves and the generation of new variants.

Given this historical perspective of the COVID-19 literature, Schwab et al. focused on the rigor of estimating the mortality rate during the first and second waves of COVID-19 in Switzerland. This study found SARS-CoV-2 in lung tissues from autopsies of deceased individuals who were not tested for the virus. The main conclusion of the study is that the COVID-19 mortality rate is likely to be underestimated. Saunders et al. then discussed the claims of an eventual causal relationship between SARS-CoV-2 infection and hearing symptoms. This study identified several problems in the published studies, such as the reliance on self-reported data, the presence of recall bias and potential nocebo effects, and the use of non-COVID-19 pseudo-control groups. Hence, claims of a possible causal effect of SARS-CoV-2 infection on auditory symptoms should be revised.

Three publications commented on issues related to SARS-CoV-2 vaccines. Günther et al. identified an inconsistency between the reported mortality rate from three published vaccine trials and the same rate predicted from the German general population. The authors also provided statistical evidence that the trials were overly optimistic about the safety/efficacy of the vaccines by focusing on total number of people vaccinated rather than the entire cohort. These authors called for improved reporting of mortality in pivotal clinical trials and for the data to be made available upon publication. Also in a clinical trial setting, Wei et al. investigated the theoretical properties of confidence interval estimation for vaccine efficacy in the presence of imperfect COVID-19 diagnostic testing. These authors also provided guidelines that are important for obtaining reliable results in terms of the respective interval estimation. In the third study, Bourdon and Pantazatos commented on the statistical errors in the calculation of the risk of myocarditis in unvaccinated and vaccinated individuals from a large-scale study. Given these statistical errors, the authors recommended that the findings of the original study should not be used to support any public health policy.

Finally, Orlando et al. identified a misstatement in the reporting of lost-to-follow-up data in a clinical trial evaluating chelation therapy. In their comment, they shared the attempt to submit an erratum and/or expression of concern and its refusal by the journal that published the original trial. Although their comment does not relate to scientific rigor in infectious disease research, it is worth noting as it might be applicable to all areas of science.

## General discussion

**Figure 1:**
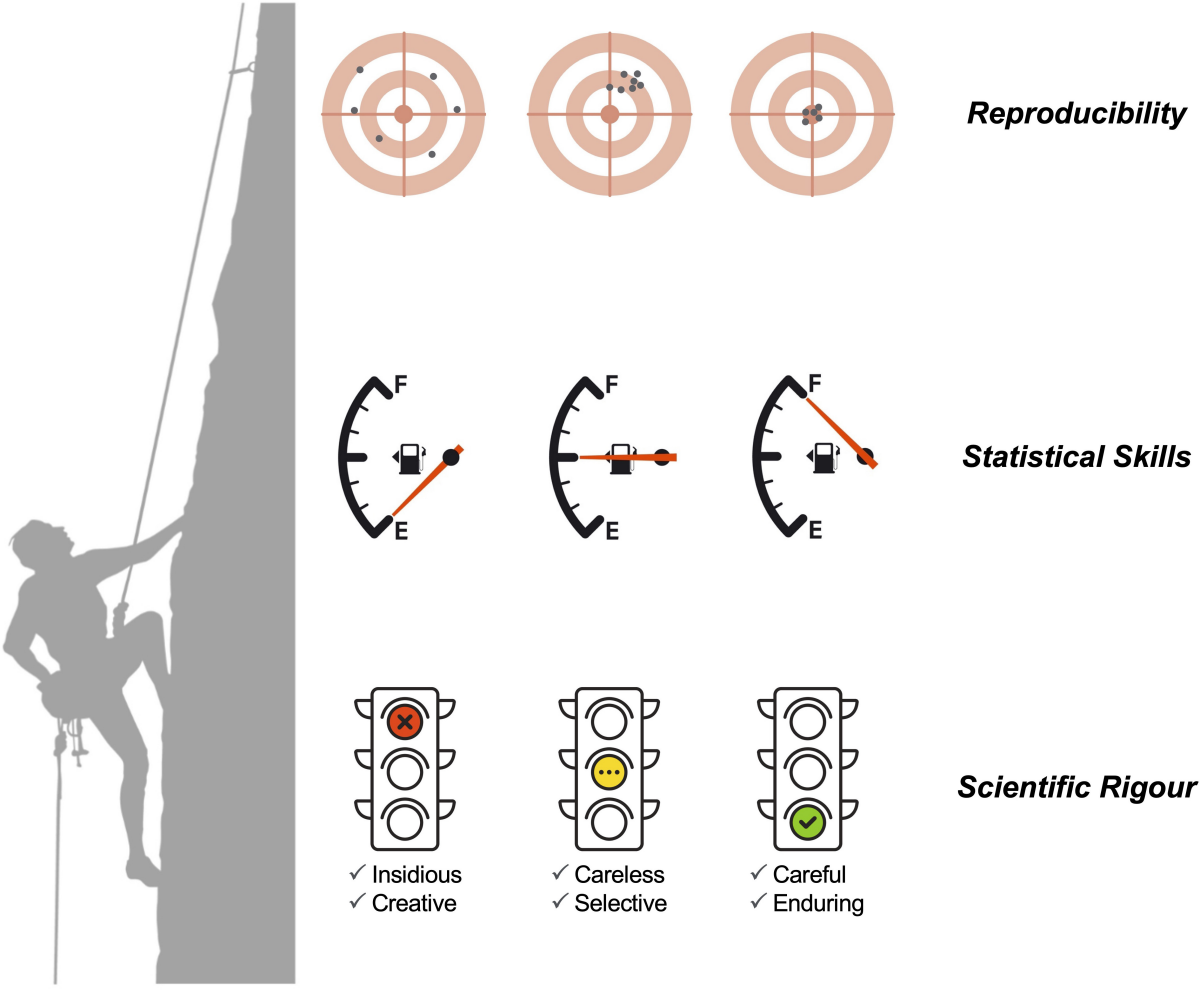
The relationship between scientific rigor (divided into six levels), statistical (and quantitative) skills, and the chance of scientific reproducibility.

The most positive aspect of this Research Topic is that all the comments did not suggest any insidious or creative rigors that could indicate ethical issues or scientific misconduct. A less positive note is the recurrent problems in terms of study design, data quality and population representativeness, and the application of sound statistical methods (13–16). We speculate that these problems result from the complexity of today's science combined with insufficient statistical skills within research teams (Figure 1). The lack of statistical skills is evident in the current research culture of consulting biostatisticians, data scientists, bioinformaticians, and mathematical modelers as a last resort (for example, when the peer-review process is already ongoing). This culture can be traced back to 1938, when Fisher famously wrote (17): “*To consult the statistician after an experiment finished is often merely to ask him to conduct a post-mortem examination. He can perhaps say what the experiment died of.”* Unfortunately, this culture is aligned with a careless rigor where a given research team randomly applies rigor only when necessary or if asked to by reviewers. It can be argued that funding limitations lead applicants to prioritize budgets for laboratory or field activities rather than data analysis. From this perspective, adherence to the highest standards of scientific rigor correlates with higher research costs and training, and thus the costs of careful and sustained rigors might only be affordable by successful institutions and research teams working in developed countries (18). This situation can be illustrated by the molecular surveillance of drug resistance in several infectious diseases. Rigorous surveillance might encompass the execution of massive sequencing efforts in a large number of biological samples, as demonstrated by studies on malaria and tuberculosis (19, 20). Researchers from developing countries might find difficult to conduct these surveillance studies with high scientific rigor on their own due to limited capacity in bioinformatics, genomics, statistics, and mathematical modeling. On the one hand, such a situation might set a strong foundation for collaborative work between researchers from developing and developed countries. On the other hand, the pursuit of a high standard of rigor might intentionally create a leadership bias toward researchers from developed countries who have the capacity and experience to conduct cutting-edge research. In this scenario, researchers from developing countries might be seen as sole data providers and secondary research players. Irrespective of this complex discussion, researchers on infectious diseases should actively seek out collaborators with more quantitative inclinations and make them real members (and not just consultants) of research teams. On the other hand, biostatisticians and related professionals might actively seek to improve their leadership skills to strengthen their collaborative and networking capacities (21). If both research communities go hand in hand, science should gain in terms of scientific rigor and reproducibility.

## Author contributions

FW: Writing—original draft, Writing—review and editing. NS: Writing—original draft, Writing—review and editing. FW and NS designed Figure 1 using images downloaded from Stock Adobe (https://stock.adobe.com/at/) under an educational institution license provided by FH Joanneum University of Applied Sciences, Graz, Austria.
